# Muscle Research and Gene Ontology: New standards for improved data integration

**DOI:** 10.1186/1755-8794-2-6

**Published:** 2009-01-29

**Authors:** Erika Feltrin, Stefano Campanaro, Alexander D Diehl, Elisabeth Ehler, Georgine Faulkner, Jennifer Fordham, Chiara Gardin, Midori Harris, David Hill, Ralph Knoell, Paolo Laveder, Lorenza Mittempergher, Alessandra Nori, Carlo Reggiani, Vincenzo Sorrentino, Pompeo Volpe, Ivano Zara, Giorgio Valle, Jennifer Deegan née Clark

**Affiliations:** 1 CRIBI- Interdepartmental Biotechnology Center, University of Padua, Padua, Italy; 2 Department of Biology, University of Padua, Padua, Italy; 3 The Jackson Laboratory, Bar Harbor, ME, USA; 4 Randall Division of Cell & Molecular Biophysics, King's College, London, UK; 5 ICGEB, Trieste, Italy; 6 EBI, Wellcome Trust Genome Campus, Hinxton, Cambridge, UK; 7 Heart Center, Georg August University, Goettingen, Germany; 8 Department of Experimental Biomedical Sciences, University of Padua, Padua, Italy; 9 Department of Anatomy and Physiology, University of Padua, Padua, Italy; 10 Department of Neuroscience, University of Siena, Siena, Italy

## Abstract

**Background:**

The Gene Ontology Project provides structured controlled vocabularies for molecular biology that can be used for the functional annotation of genes and gene products. In a collaboration between the Gene Ontology (GO) Consortium and the muscle biology community, we have made large-scale additions to the GO biological process and cellular component ontologies. The main focus of this ontology development work concerns skeletal muscle, with specific consideration given to the processes of muscle contraction, plasticity, development, and regeneration, and to the sarcomere and membrane-delimited compartments. Our aims were to update the existing structure to reflect current knowledge, and to resolve, in an accommodating manner, the ambiguity in the language used by the community.

**Results:**

The updated muscle terminologies have been incorporated into the GO. There are now 159 new terms covering critical research areas, and 57 existing terms have been improved and reorganized to follow their usage in muscle literature.

**Conclusion:**

The revised GO structure should improve the interpretation of data from high-throughput (e.g. microarray and proteomic) experiments in the area of muscle science and muscle disease. We actively encourage community feedback on, and gene product annotation with these new terms. Please visit the Muscle Community Annotation Wiki .

## Background

Technical innovations in recent years have enabled the production of vast amounts of scientific research data, using a variety of methods, and covering many different species. These innovations provide the opportunity for evaluation of large datasets to generate and/or support novel hypotheses. In dealing with this wealth of data, scientists are held back by differences in technical language among research communities, compounded by the absence of computable information on the relationships between biological processes.

An example of linguistic ambiguity within the muscle biology community is seen in the use of the word 'plasticity'. This word could mean the quality of adaptability, but is often used to indicate the process of adaptation. In addition to complicating the work of research scientists, such ambiguity also presents real difficulties for those who wish to write data mining software. Such software attempts to automatically handle information about the relationships between biological processes and between gene products. There is a particular need for good data mining software in high-throughput work, which is a prominent part of current muscle biology research. The aim of the Gene Ontology (GO) project [1,2] is to provide a standard language for the description of gene products, thus enabling scientists and software engineers to resolve language problems.

To provide this standard language, the GO project is developing ontologies and using them in annotation of gene products. There are three non-overlapping ontology domains, so that gene products may be categorized according to GO terms representing the molecular functions they carry out (using the Molecular Function ontology), the cellular locations where they act (using the Cellular Component ontology), and the biological processes in which they take part (using the Biological Process ontology). The three ontologies are separate, but within each ontology the GO terms are related to one another. These relationships indicate where one category is a part (*part_of *relationship) or type (*is_a *relationship) of another category, or where one category regulates (*regulates*, *positively_regulates*, or *negatively_regulates *relationships) another category. For a more comprehensive explanation see [3]. Each ontology can be used as a standard terminology to facilitate a biologically meaningful description of the roles of genes and their products in any organism. Gene products can be annotated to any number of GO terms within one or more of the ontologies to capture information about their various roles within these given domains. The Gene Ontology has for several years included a number of terms describing muscle biology, and the GO has already been used extensively for statistical data analysis in muscle biology studies. For example, the GO was used in an analysis of the global transcriptional changes that take place in skeletal muscle in relation to estrogen status [4], and in an expression profiling study of the transcription factor MyoD during myogenic differentiation [5]. However, to fully support the current needs of the muscle research community, especially with respect to the study of disease, a considerable expansion of the terms relevant to muscle biology is required. We describe here an effort to improve the structure of muscle terms in the GO biological process and cellular component ontologies. The work was carried out as a collaborative project that brought together the GO Consortium, the Genomic Research Group of CRIBI Biotechnology Center at the University of Padua, and several research groups involved in muscle biology. We sought to improve GO terms that would specifically support muscle biology research in areas relevant to the investigation of muscle-related disease. By bringing together muscle and ontology experts, the GO structure was systematically improved in five areas: muscle contraction, plasticity, development and regeneration; and for cell regions in the sarcomere and membrane-delimited compartments.

## Methods

Following the example of the Immunology Content Meeting [6] and of other GO ontology development meetings, the muscle-related GO Content Meeting brought together experimental biologists and ontology developers to define the terms relevant to this specific research field.

Content-oriented meetings facilitate large-scale changes in specific areas of the Gene Ontology. A content meeting is usually organized as a multi step process. The first steps are normally done by the ontology developers, gathering information related to the field of interest from books, reviews and scientific papers and organizing it into an ontological format. The ontology is finally presented to the experts in the field during the content meeting, to be discussed and refined. The work described in this paper was carried out using a rather different approach. The initial steps were mainly carried out by the muscle biology research community, while the final discussion and refinement was carried out during a meeting with invited ontology developers. This approach was possible because a member of the muscle community (EF) spent six months working in ontology development and annotation at the GO editorial office and gained further experience by taking part in a previous GO content meeting. She then rejoined her research community and led the ontology development effort. Throughout the initial community editing process, GO Consortium editors provided technical assistance and advice on representation of language by means of frequent web-based ontology editing meetings. Following this editing phase, ontology developers from the GO Consortium were invited to meet with the group of domain experts for further discussion and revision of the structure of the GO. This two-day meeting was entirely devoted to live editing the GO, during which further changes were made to its structure and content. The community-based ontology development model was extremely positive and productive, as it enabled the lead ontology developer to access a wide range of domain experts, mostly locally available, and all with cutting-edge knowledge of the field.

At the end of October 2007, the changes were incorporated into the GO, and are now available for all GO users.

In addition to creation and improvement of GO terms, cross-references were made with a number of other resources. The Adult Mouse Anatomical Dictionary [7] was used as a source of definitions and ontological structuring for muscle contraction anatomical terms, and the Cell Ontology [8] was consulted for cell type definitions. The resulting terms were cross-referenced to these other ontologies and, where appropriate, new definitions were contributed to the other ontologies. For example, a new definition of the satellite cell type was created and introduced in both the Cell Type and GO ontologies, and the resulting terms were cross-referenced. Gene Ontology term names are given in bold in this text.

## Results

The muscle research community, in collaboration with the GO Consortium, has completed an initiative to greatly expand the muscle biology representation in the GO biological process and cellular component ontologies. The work focused on improving and adding terms urgently needed for current priority areas in research.

The main focus of the work was skeletal muscle, with specific consideration given to the processes of muscle contraction, muscle plasticity, muscle development and regeneration; and to the sarcomere and membrane-delimited cell compartments. Our aims were to update the existing structure to reflect current knowledge, and to resolve in an accommodating manner, the ambiguities in the language used by the muscle community. This collaborative effort drew on the knowledge of an extensive community of muscle experts, and resulted in the addition of 159 new terms and the improvement of 57 existing terms.

These different areas of muscle biology were addressed to support specific research needs. In the following text, the motivation for the changes and the details of each set of changes are described.

### Muscle Plasticity

There are two different possible biological meanings of the commonly used phrase 'muscle plasticity', such that plasticity could be either the quality of adaptability or the process of adaptation. In ontology development it is essential to be clear about which term represents which process; and to ensure that the language is unambiguous, whilst still reflecting community usage. The existing **muscle plasticity **term was ambiguously named, risking incorrect use in annotation or text mining. However, as the term was clearly defined to describe the process of adaptation, we were able to resolve the problem by renaming the term **muscle adaptation **(leaving muscle plasticity as a related synonym, to help researchers find the term). This action resolved the ambiguity, but accommodated the common uses of the word 'plasticity' in domain literature by retaining the word as a searchable related synonym.

**Figure 1:**
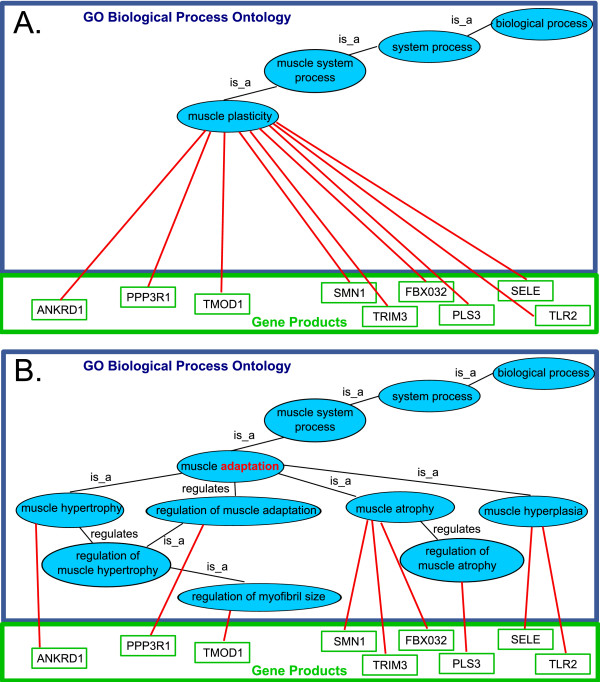
**Muscle plasticity GO node**. As an example, the process 'muscle plasticity' is shown before (Panel A) and after (Panel B) our modifications. Previously, the process of muscle plasticity had no specific child terms, therefore all annotations of the gene products involved in subprocesses of muscle adaptation had to be made directly to the muscle plasticity term. As part of our work we renamed muscle plasticity to muscle adaptation (highlighted in red) in keeping with the existing term definition, and added new terms for various sub-processes of muscle adaptation such as muscle atrophy, hypertrophy and hyperplasia. As a result of this work the gene products previously annotated directly to muscle plasticity can now be annotated to the more specific process terms giving far better reasoning power for analysis of high-throughput experiments. GO terms are in blue ovals, and annotated gene products are in green rectangles. Black lines marked 'is_a' indicate the is_a relationship. Black lines marked 'regulates' indicate the regulates relationship. Red lines indicate annotation of a gene product to a GO process term. Annotation of a gene product to a GO process term indicates that the gene product participates in the process represented by the GO term.

There are many stimuli that bring about muscle adaptation. Musculo-skeletal adaptability studies include examination of a muscle's response to joint immobilization, spinal cord injury, electrical stimulation, chronic stretch, exercise-induced injury, and microgravity. Adaptive events that occur in muscle fibers and associated structures (motor neurons and capillaries) include **atrophy**, **hypertrophy**, and **hyperplasia**, and these involve alterations in regulatory mechanisms, contractile properties, fiber-type compositions, and metabolic capacities. Previously these processes were not covered in the GO as the term **muscle plasticity **(Figure 1A) had no more specific child terms. During the editing work, terms covering these important sub-processes have been included (Figure 1B).

Using these new more granular terms, biologists will be able to annotate gene products in more detail. Prior to our work, if a gene was thought to be involved in muscle atrophy, the user had only the option of annotating directly to the general term 'muscle plasticity'. As a result of our contribution, the ontology now includes child terms representing muscle atrophy, hypertrophy and hyperplasia. It also includes generic regulation terms under each of these processes, and under these regulation terms the actual regulatory processes are grouped. To illustrate the advantage of creation of these new terms the muscle experts have contributed some annotations that could previously only be made to the general **muscle plasticity **term (Figure 1A) and that can now be distributed amongst the more specific child terms for much greater reasoning power (Figure 1B). This small amount of annotation clearly shows how much better this enhanced structure is for distinguishing sets of gene products involved in the various processes that contribute to the general process of muscle adaptation. Though we have shown only a handful of gene products, it can easily be imagined how much more powerful the system will be in automated analysis of the activity of thousands of gene products, as is the case in a microarray experiment. For example, once the relevant gene products are fully annotated, it will be possible to detect by microarray experiment those stimuli that upregulate hundreds of genes involved in muscle hypertrophy, whilst barely affecting the regulation of genes involved in muscle atrophy.

This new set of terms should assist in the annotation of gene products involved in the control of muscle fiber-type diversity, providing potential new targets for the treatment and prevention of different disorders ranging from metabolic to neuromuscular diseases, for example Type 2 diabetes and muscular dystrophy [9]. We have explained this example of muscle plasticity very fully to illustrate the motivation behind our ontology development work. The work carried out on other areas of the ontology will bring similar benefits with regard to other critical areas of research, and we describe these pieces of work somewhat more briefly below with reference to the areas of research that they are intended to support.

### Muscle Contraction

The definition of the term **muscle contraction**, which previously existed in the GO, has been considerably improved and all of its descendants have been reorganized. The new structure represents several forms of muscle contraction and their relationships with the various types of muscle. To reflect this, there is also a greatly expanded set of terms describing the different contractile capacity of muscle. Striated muscle contracts and relaxes in short, intense bursts, whereas smooth muscle sustains longer or even near-permanent contractions. This difference was captured by the creation of is_a children, **phasic smooth muscle contraction **and **tonic smooth muscle contraction**, under the parent term **smooth muscle contraction**. Since the process of smooth muscle contraction varies with the anatomical location of muscles, terms such as **vascular muscle contraction **and **gastro-intestinal muscle contraction **were also created.

Muscle contraction is actively regulated by a series of events, for which appropriate regulation terms have been added. These include several processes such as cross-bridge formation, cross-bridge cycling, and filament sliding, which are necessary for force generation during muscle contraction. Multiple molecular components, such as sarcoplasmic proteins, have a role in regulating the muscle contraction. For instance mutations in several Z-disc proteins in the sarcomere, that are important for the cross-linking of thin filaments and transmission of force generated by the myofilaments, have been shown to cause cardiomyopathies and/or muscular dystrophies [10]. To accommodate this, a definition of the sarcomeric **Z-disc **has been added to the component ontology and extended to include recently discovered novel attributes associated with this structure, such as mechanosensation and mechanotransduction, thereby allowing users to view the Z-disc not so much as a static, but now as a flexible structure with important implications for signal transduction as well [11].

### Calcium signaling

**Figure 2:**
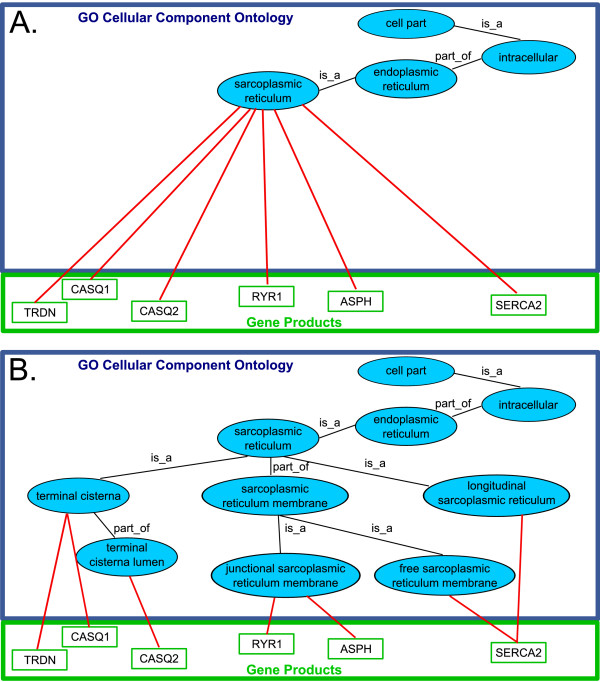
**Sarcoplasmic reticulum GO node**. As an example, the cellular component 'sarcoplasmic reticulum' is shown before (Panel A) and after (Panel B) the modifications described in this paper. Previously, the term sarcoplasmic reticulum had no specific child terms, therefore all annotations of the gene products known to act in specific regions or sub-types of the sarcoplasmic reticulum had to be made directly to the parent term. As a part of our work we added new terms to describe various regions and sub-types. As a result, the gene products previously annotated directly to sarcoplasmic reticulum can now be annotated to the more specific child terms giving far better reasoning power for analysis of high-throughput experiments. Black lines marked 'is_a' indicate the is_a relationship. Black lines marked 'part_of' indicate the 'part_of' relationship. Red lines indicate annotation of a gene product to a GO cellular component term. Annotation of a gene product to a GO cellular component term indicates that the gene product acts in the cellular location represented by the GO term.

The previously described process of muscle plasticity is closely linked with, and highly dependent on, calcium metabolism and transport, as muscles use calcium ions as their main regulatory and signaling molecule. Therefore, calcium ion-dependent processes control the properties of the mechanisms of contraction and relaxation in different types of muscle fibers [12]. The sarcoplasmic reticulum (SR) is a sub-compartment of the endoplasmic reticulum (ER) and is molecularly specialized for calcium release, uptake, and storage and for the contraction-relaxation cycle in skeletal muscle fibers [13]. Recognizing the importance of this, we focused part of our work on improving the existing terms describing sarcoplasmic reticulum and its role in regulating the calcium ion-dependent processes. Terms such **regulation of skeletal muscle contraction by calcium ion signaling **and **regulation of skeletal muscle contraction via modulation of calcium ion sensitivity of myofibril **were added as part_of children of **muscle contraction**. In addition, the sarcoplasmic reticulum compartment and its components are covered by an expanded hierarchy of terms. Whilst the term **sarcoplasmic reticulum **pre-existed in the GO (Figure 2A) we have been able to add many new child terms (Figure 2B). This allows the gene products whose locations of action could previously only be categorized loosely using the single **sarcoplasmic reticulum **term (Figure 2A) to be categorized in far more detail (Figure 2B). We give this example in detail with annotations to indicate how the additions to the cellular component ontology provide similar benefits to those in the biological processes ontology, previously illustrated by use of the **muscle plasticity **example. The new child terms in this case include **longitudinal sarcoplasmic reticulum**, **terminal cisterna**, **terminal cisterna lumen**, **free sarcoplasmic reticulum membrane**, and **junctional sarcoplasmic reticulum membrane**. These new GO terms will aid our understanding of normal muscle processes and muscle pathological conditions such as dystrophinopathies, Brody's disease, and malignant hyperthermia. These have been shown to be due to alterations in calcium ion-dependent ion channel activities [12].

### Muscle Types

Muscles can be divided into striated and smooth types. Smooth muscle or 'involuntary muscle' is found within structures such as the oesophagus, stomach, intestines, bronchi, uterus, and blood vessels. Unlike skeletal muscle, smooth muscle is not under conscious control. Cardiac and skeletal muscles are striated in that they contain sarcomeres and are packed into highly regular arrangements of bundles.

Skeletal muscles are further divided into two subtypes, slow-twitch and fast-twitch muscle, depending on their contractile capacity. The biology of these two muscle types is key in current research, so we worked to represent it correctly as part of the biological process ontology. Improvements were made to the representation of these areas, to ensure that the usage of the words 'skeletal' and 'striated' was representative of that in the community. Importantly, these terms were also cross-checked by a cardiovascular physiology community group, whose ontology development effort took place at the same time, and which also touched on voluntary/involuntary muscle processes (David Hill, personal communication).

### Muscle Development and Regeneration

Myofibers, the functional unit of skeletal muscle, are long cylindrical multinucleated cells that vary in their morphological, biochemical, and physiological properties. They are derived from myoblasts: cells committed to the skeletal muscle lineage. Upon fusion, myoblasts form myotubes, which are further remodeled into myofibers [14]. The **skeletal muscle development **subtree has been enhanced during our work with a new hierarchy of terms describing **myoblast**, **myotube**, and **myofiber development**, and the mechanisms of their regulation. To accommodate recent data, a distinction was introduced between head and trunk muscle development [15].

Many terms have been added to cover the process of cell regeneration and its regulation in skeletal muscle tissue. These include terms such as **satellite cell activation involved in skeletal muscle regeneration **and **satellite cell compartment self renewal involved in skeletal muscle regeneration**. Satellite cell processes are considered particularly important, since their activation is involved in muscle regeneration. Satellite cell proliferation, differentiation, and self-renewal are essential for proper myofiber turnover; an ongoing process that maintains proper muscle tissue viability [16]. Moreover, in adult skeletal muscle, the self-renewing capacity of satellite cells contributes to muscle growth and adaptation [17]. Skeletal muscle is capable of complete regeneration due to the presence of stem cells that reside in skeletal muscle and non-muscle stem cell populations. However, in severe myopathic diseases such as Duchenne Muscular Dystrophy, this regenerative capacity is exhausted [18]. We have attempted to support research into these areas by addition of the relevant terms.

## Conclusion

We have described an ontology development effort that provides a valuable resource for functional annotation of gene products related to muscle biology. New terms supporting critical research areas are now available, and existing terms have been improved and reorganized to reflect their usage in muscle literature. There are a number of important advantages to a research community in having their field accurately represented in the GO. Our revised ontology structure should facilitate the interpretation of high-throughput experiments (e.g. gene expression microarrays) in the areas of muscle science and muscle disease. Such studies yield a very large number of data points, so that investigation of how genes specifically contribute to a disease phenotype is challenging [19]. However the use of GO ontologies and annotations in statistical analysis should greatly simplify this [20].

Obviously, a critical component of such analysis is the comprehensive annotation of relevant gene products. To enable community annotation, we have provided a Muscle Biology Community Annotation Wiki . The wiki contains editable annotation pages for 172 genes associated with muscle development and function.

Users can review existing Gene Ontology annotations for any gene of interest, and add information about any aspect of the biology of a gene from any species. They can also contribute annotations of gene products involved in muscle biology to all GO terms, thereby supporting the next step in research into these critical areas of muscle biology.

## Competing interests

The authors declare that they have no competing interests.

## Authors' contributions

EF is a PhD student working under the supervision of JD for this GO project work. They collaborated together throughout the process, devising and implementing the new system of community-driven ontology development. EF worked with the muscle community under the supervision of GV, whilst JD provided liaison with the GO Consortium. EF and JD prepared the manuscript for publication. GV provided the initial idea and the funding for the community content meeting, and revised critically, and gave final approval on the manuscript.

The Muscle Biology Working Group includes the muscle biologists and GO Consortium ontology developers who contributed their time and expertise to the content development effort. The members of the working group were: Stefano Campanaro, Alexander D. Diehl, Elisabeth Ehler, Georgine Faulkner, Jennifer Fordham, Chiara Gardin, Midori Harris, David Hill, Ralph Knoell, Paolo Laveder, Lorenza Mittempergher, Alessandra Nori, Carlo Reggiani, Vincenzo Sorrentino, Pompeo Volpe, Ivano Zara.

## Pre-publication history

The pre-publication history for this paper can be accessed here:


